# Choosing a medical school: Advice for applicants

**DOI:** 10.15694/mep.2020.000156.1

**Published:** 2020-08-04

**Authors:** Richard Hays

**Affiliations:** 1 James Cook University

**Keywords:** medical school selection, primary medical education, career choice

## Abstract

This article was migrated. The article was marked as recommended.

Applying for a medical school place is a challenging step that can have long term consequences. Competition is strong, both for applicants to gain a place and for medical schools to recruit the ‘best’ applicants. Strategies to increase candidate success flourish, but many are unproven. Potential applicants should invest time and effort in exploring which schools and programs might suit best their particular circumstances and interests. Back-up plans should be considered for both success and failure. Not succeeding first time or at a first-choice school may be disappointing, but there are other paths to success in both medical and non-medical careers. Before accepting an offer, make sure that this is the best choice for the best reasons, and then make the best of whatever follows. In the longer term, career success is shaped more by what happens after than during primary medical education.

## Introduction

Applying for a medical school place is a big step, a ‘fork in the road’ of life. The outcome, either way, may have far-reaching impact. Success begins the transition to becoming a medical student, a major event that for most spans the emergence into mature adulthood. Embarking on a medical career requires strong motivation, because for many this is the start of a lifelong commitment to continuing education throughout a medical career that can last 30-40 years. Lack of success may bring disappointment and soul-searching.

How should applicants prepare for this major life event? The application process may be difficult, requiring evidence of academic and other information, through a highly competitive process that can be stressful. Many regard the application as the goal of several years preparation, but is it the beginning of an end or the end of a beginning? Does the medical school choose the applicant (like the sorting hat at Hogwarts) or do applicants choose the medical school? Success in the application and beyond may depend on the planning and effort invested early. Studying medicine will dominate life for the next 3-6 years but is only the first of many career stages.

Many potential applicants think of the application process as a simple dichotomous process: ‘yes’ is great, ‘no’ is terrible. But is that necessarily the case? Back-up plans should be made for both outcomes. Applicants should think through: ‘What if I get an offer at a medical school I know little about or may not like? Does it matter which medical school I attend? Can I afford to leave home and family support for years of study? How can I increase my performance in selection tests? What if I do not receive any offers?’

Most applicants will apply to more than one medical school. In some countries the number of choices may be limited through national processes, but in all cases each application can be hard work and attending interviews at several different schools can be logistically difficult and costly. It is therefore important to think carefully about which and how many medical schools to apply to, based on your own interests and strengths. There will be plenty of advice from family, friends, school counsellors and social media, but it would be wise to do some personal research to inform your decisions. Search widely and avoid making decisions too early. There is unlikely to be a single right answer for all applicants. What was right for a relative or friend might not be right for you.

This paper summarises the advice I have often been asked to provide to potential applicants for a medical school place. Sources are my international experiences in academic and leadership roles, managing selection processes, training, employing and working with graduates from many medical schools, and accrediting medical programs. It is only one view, but the information may be a useful guide for potential applicants for what to consider when applying to study medicine.

## Considerations

### Reputation

Most of the world’s early cultures developed and passed on health care practices and so medical education has existed in some form for a very long time. The first European medical school may have been the Asklepion on the island of Kos, run by Hippocrates and his followers. The re-emergence of Christianity in about the 11-13 ^th^ centuries resulted in the establishment of Universities with a ‘Papal Bull’, or official church recognition. These universities usually offered theology, philosophy and medicine. The earliest were in Bologna, Montpellier, Vienna, Prague, Paris, Oxford and Cambridge. These were rather theoretical, philosophy-driven programs with a strong twist of religion, while most health care was delivered by lesser-trained but more practical midwives, apothecaries and barber-surgeons. Similar schools were established in Persia and the Arab world and for several centuries leading physicians in the three monotheistic religions shared knowledge and experience. More practical, workforce-aligned schools were established in northern Europe, such as the school at Leiden. The shape of current medical education emerged about 200 years ago, moving from apprenticeship to class-based, became more standardized about 100 years ago (Flexner, 1911), and over the last 50 years has evolved further as knowledge expanded rapidly and new learning technologies emerged. Many of the well-established schools are very strong performers in medical research and are at the forefront of advances in medicine.

While that is interesting (at least I think so), is a medical school in a University that is 800 years old better now than one that is much more recent? Is a medical school that is in the top (say) 100 in international research performance, or has been the base for Nobel prize winners, a better place to study for a primary degree in medicine? There is no evidence for either assertion, but it is part of the human condition to be attracted to brands and reputation. Attending these schools can be a great experience, but so can attending lesser known schools. Medical education is now a very different experience that requires specific expertise that is not related to history or research expertise.

**Tip 1.** Applying to the schools with strong reputations is fine, but do not be worried if you get offered a place in a lesser known school. In the long run, this is probably of little importance.

### Mission and stated preferred outcomes

Many mission statements are rather bland assertions about being ‘world class’ and promoting ‘excellence’. Often these are written by public relations people and may explain very little about a university or school. Gaining an understanding of the student experience at prospective schools is important, because aligning values could be an important step when committing 3-6 years of your life to a particular institution. While being part of a medical school class is a kind of ‘bubble’ that lasts for the duration of the program, sometimes with little contact with the rest of the university, there is an institutional ‘culture’ in which that bubble exists. It may be better to choose a culture in which you feel comfortable. Examples of cultural aspects include strong support for students; concern with helping under-served communities, striving to solve major technical/scientific challenges (e.g. ‘curing cancer’), and highly altruistic student organisations. The concept of ‘social accountability’ is emerging as an alternative to the traditional ‘technocrat’ model ( Boelen, Dharamsi and Gibbs, 2012). One under-recognised effect of cultural immersion is the potential for role modeling: students may be more likely to follow in the footsteps of their teachers and adopt the institutional culture. While cultural aspects may be reflected in mission statements, it is wise to look more deeply by speaking with current students. Seek out several views, as the loudest voices may not necessarily represent the whole truth.

**Tip 2.** Aligning values may be important.Look beyond mission statements and course outlines to explore the culture of the school. Current students may be the best source of information.

### Marketing and competition

Most medical schools do not advertise for applicants, because demand usually exceeds the number of places, but medical schools can be very competitive. The competition lies in attracting ‘the best’ applicants that suits the self-image of the school. For the more ‘elite’ schools, characteristics of the ideal medical school applicant may include: at the very top of prior academic achievement; prior attendance at the ‘right’ school, college or university; leadership roles in previous educational or career stages; excellent at music and/or sport; and an ambition to be at the top of a narrow medical or surgical specialty with strong research interest. Schools with a mission involving primary care or helping under-served populations may prefer ‘broader’ applicants from more diverse backgrounds with an interest in people and social justice, accepting slightly lower (but still very strong) prior academic performance. However, these are caricatures that may be more perception than fact, as all medical schools accept all kinds of applicants, generally believing that diversity is good. Many elite schools, both public and private, offer financial support for applicants less able to self-fund and some actively target applicants from minority and underserved populations and communities.

**Tip 3.** Look beyond any obvious marketing to get a sense of what each school is looking for in applicants and is offering as support.

### Location

While attending a local medical school and living with family is often less expensive and more convenient, emerging as an independent, responsible adult can be easier if living away from home. There are cultural influences here. In the UK and North America, leaving home to attend university appears to be a rite of passage. In Australia, the distances between medical schools are so large that most applicants prefer to be closer to home. Some live too far from medical schools to travel daily, for others this issue may be just a choice. If finance allows mobility and the applicant is resilient enough not to need close family support, then location should not matter. It may be unwise to accept an offer elsewhere if it means regular commuting to see partners and children, or if it requires substantial paid work to make this affordable: both are difficult to fit in and can result in poor academic performance. Further, graduation with substantial debt may influence career choice ( Hays *et al.*, 2015).

**Tip 4.** Decide early whether you have the funding and ability to thrive after a big move away from family and friends.

### Recognition within a jurisdiction

Medical schools and universities are usually highly regulated and must be approved by responsible authorities. Many schools fall within the remit of national or regional organisations that accredit medical programs. This accreditation process is based on regular (6-10 yearly) reviews of programs from the perspective of governments, medical licensing authorities, specialty training organisations, employers, patient groups, recent graduates and current students. The better known and longer standing accreditation authorities are the General Medical Council (GMC) in the United Kingdom, the Liaison Committee on Medical Education (LCME) in North America and the Australian Medical Council (AMC) in Australia and New Zealand ( General Medical Council, 2015; Liaison Committee on Medical Education, 2020; Australian Medical Council Limited, 2012). There are many others, most with similar standards based on those of the World Federation of Medical Education (WFME) ( World Federation of Medical Education, 2015). All local graduates recognised as medical practitioners must have attended a medical school accredited within the local jurisdiction. This includes schools that are centuries old right through to brand new schools, which must have approval to commence. Degrees from all medical schools accredited within a particular jurisdiction are recognised by every employer and specialty training program within the same jurisdiction and all applicants from locally accredited medical programs should be treated equally. The websites of the accreditation authorities list all approved programs, so this is easy to check.

**Tip 5.** So long as you attend a medical school that is approved by the local accreditation authority, the degree is of equal status to those from all other accredited schools within the same jurisdiction.

### Recognition outside of the home jurisdiction

Attending a medical school outside of your home country may be much more complicated. While this can be a great personal experience, international medical programs are generally not recognised by national accreditation authorities in your home country. This means that graduates may have to go through procedures for assessing international medical graduates even though they have an inalienable right to return ‘home’. This is not necessarily a difficult academic task - many programs are very good and have similar standards to local programs - but this path can be expensive and time consuming. There are some exceptions, such as locally accredited medical programs managed and delivered in some other countries, but these represent only a minority of cases. Applicants choosing this path should seek advice from the local accreditation authority, rather than rely on advice from the medical school. A list of recognized medical schools is available for checking ( Foundation for the Advancement of International Medical Education Research, 2016). The situation is subject to change, so advice at entry may not be correct at graduation. It may be best to regard attending an international medical school as an enriching life option with potential emigration outcomes rather than equivalent to a locally accredited degree. When not receiving an offer of a place in a local medical school, boosting local qualifications and re-applying may be a’ safer’ option than accepting an international offer

**Tip 6.** Be cautious about applying to a medical school in a jurisdiction outside of the country where you live. If the goal is to return home for work in clinical medicine, there may be a complex pathway to success.

### Learning experience

While graduate outcomes are usually agreed within each jurisdiction and are similar across international boundaries, the structure of the curriculum and assessment practices may vary substantially. It is safe to assume that learning at medical school will be very different to learning at secondary school or in general college and university degrees. More traditional programs include many lectures, with the teachers providing strong direction about what to learn when and in what sequence, with linear modules that may be assessed independently. Other programs are integrated, with more self-directed learning based on clinical scenarios,so that students have more influence over sequencing and depth of learning in certain areas. One version of the latter is problem-based learning (PBL), which for some reason is unpopular with some (a ‘teach yourself’ curriculum), although popular with others ( Hays, 2008). Some programs introduce clinical work quite early, which adds interest and may improve understanding ( Yardley *et al.*, 2013). Assessment can vary from large written tests of factual knowledge to the use of many more methods to capture information about learning progress across the breadth of a curriculum. Most programs take a ‘hybrid’ approach, using many different learning and assessment approaches, but a few programs are at one or other extreme ends of the spectrum. New or recently renewed programs may engage learners more in discussion about how best to learn and may be more innovative. There is no evidence of any longer-term differences in graduate outcomes, but more interactive, small group programs seem to bring more short-term enjoyment to some students ( Colliver, 2005). This is about personal learning style preferences: should you have a strong preference for any particular program structure, explore this by talking with current students.

**Tip 7.** Most medical programs have similar structures and processes, although some are at the extremes of the spectra from teacher-driven to student driven, modular and linear to integrated, and narrower to broader assessment practices. Applicants with a strong preference should explore the learning experience at a medical school prior to applying.

### Selection processes

Most medical schools base offers on a range of academic and personal qualities measurements. While prior academic performance best predicts the next academic performance, a medical career is more of a marathon than a sprint and other things are important to sustain careers. Many medical schools use a variety of non-academic measures in calculating a rank order for offers. Some use ‘aptitude tests’, which are controversial because it is not certain just what these measure and test scores are very weak predictors of success. Situational judgement tests may be more useful and are being adopted more widely ( Patterson *et al.*, 2017). These maybe a novel experience for many applicants so gaining familiarity is wise. Interviews are commonly used and often predict performance at graduation or later: structured panel interviews and Multi Mini Interviews (MMIs) are the most commonly used ( Rees *et al.*, 2016). Testimonials and personal statements seem to contribute little and are open to falsification. All non-academic measures are open to coaching, although the benefits of coaching are thought to be small. It may be better to be yourself, rather than a contrived medley of coachable attributes that may even reduce likelihood of selection. Practising interviews with family and friends may help reduce anxiety. Where possible, individualise applications, as each school may require different information or emphases and interviews provide opportunities to show that you know about the ‘this’ program. Because different medical schools use different measures (and apply them differently), the influence of each selection method at each school is difficult to determine. However, applicants with an aversion to one measure may prefer applying to a school that does not use that measure. All necessary information is on medical school websites. Monitor, but do not believe blindly, social media and other non-verified sources of information.

**Tip 8.** Explore carefully what measures of academic and personal qualities are used in determining offers at each school being considered. It may be better not to apply to a school using selection methods with which you are uncomfortable. The benefits of attending paid coaching courses in selection methods, such as aptitude tests and interviews, are unproven.

### Take the first step first

At least initially, put aside any future specialty preference, even if you have a strong interest in a particular career pathway. Instead, focus on what will get you to the point where specialty training is applied for. During the primary medical training phase, explore and try to enjoy all curriculum components and placements. All are relevant to most careers and may influence career choice and enjoyment. Graduation from any accredited primary medical program confers eligibility to pursue a career in any specialty. The next stages have their own selection processes and doing your best during the primary medical degree may strengthen your application and readiness for the next stage.

**Tip 9.** Defer making a firm decision about ultimate career choices until the end of primary medical training. Enjoy what this program offers and explore options.

### Reflect: Am I really suited to a medical career?

Before applying, and again should an offer not be received, pause to reflect on whether this is the right career decision. Some people apply for a medical school place for the wrong reasons. Having academic strengths is not on its own enough. Having relatives in medicine does not necessarily provide motivation. Medical practice can be hard work, place your life at greater risk and cause great stress for both yourself and those close to you. The knowledge base is growing exponentially, practice changes continuously and staying current over a career requires hard work. While the professional status is generally high, making mistakes can have tragic consequences for all. It is not usually a path to wealth: most doctors earn comfortable rather than high incomes. There are some indications that people attracted to status and high income do not find their careers as rewarding. It may be wise to do either paid or voluntary work in the health care system to strengthen understanding of what might lie ahead.

**Tip 10.** Before applying, and certainly before accepting a place, check that this is the right career choice. Explore what a medical career is really like and be honest with yourself. If in any doubt, talk to someone outside of the immediate family. For some, not being offered a place may be the best outcome, re-directing efforts to other careers that may better suit.

### Accept the outcome and make the best of the opportunity

Most applicants will receive one offer or accept the first offer they receive. This is an important decision, so do not rush. However, once the decision to accept an offer is made, embrace it with enthusiasm! This is one of the nicest life stages transitions you may have. Most students enjoy the experience - a lot of life ‘happens’ while at university - and students become part of the culture and history of their medical school. We defend our programs, form strong bonds with classmates and are proud of our alma mater for life.

**Tip 11.** After accepting an offer, make this decision the right decision. Be happy, grab new experiences and make the most of the opportunities.

### Avoid accepting a less desirable offer, planning to switch medical schools

Some applicants accept a place in a medical school that is not really the desired option, hoping to switch medical schools next year. However, switching can be very difficult, for three reasons. The first is that curricula often vary substantially and so students at the end of Year 1 may be at a very different place in their learning to those at other medical schools. Second, applying for a swap to a medical school to which a previous application was not successful raises questions about fairness. If you missed out last year by 10 places, and apply to swap into the same cohort at that school, then should the medical school call up the other 9 and offer them a place too? Third, funding can be complicated, such that in some nations medical schools receive ‘block’ funding for producing an agreed number of graduates. Therefore, they may not receive any additional funding for an extra student, although will incur additional costs.

**Tip 12.** Apply only to medical schools that you could happily spend 3-6 years of your life. Switching medical schools is very difficult due to curriculum diversity, perceived fairness and funding models.

## Summary

Commencing medical studies is a major event in life and should be approached with careful consideration of the options and what might be best for individual applicants. The stakes for applicants are high, because competition is strong and the outcomes long term. Strategies to increase success flourish, but many are unproven. Back-up plans should be considered for both success and failure. Many more apply than can succeed with their first choice but, on the other hand, many more receive an offer at another school or find another path that gets to the desired outcome eventually. Other career options can provide rewarding professional lives and should be considered. Before accepting an offer, make sure that this is the best choice for the best reasons, and then make the best of whatever follows. In the longer term, careers are shaped more by what happens after than during primary medical education.

## Take Home Messages

• Applying for a place in an undergraduate medical program is a big step that requires careful thought, research and planning.
• Each medical program uses different information in and applies scores differently in determining rankings for offers.
• Invest in understanding what each program offers and individualise applications.
• Be wary of paid courses that claim to increase chances of selection, as there is little evidence that this correct.
• Sometimes not receiving an offer is the right decision that helps you find other satisfying career pathways.

## Notes On Contributors

Richard Hays, MD, PhD, is an experienced medical educator who has had several leadership roles in Australian and UK medical education, including responsibility for selection of incoming medical students. ORCID: https://orcid.org/0000-0002-3875-3134

## Declarations

The author has declared the conflicts of interest below.

For transparency, I am the Editor-in-Chief of MedEdPublish, but this is a personal view.

## Ethics Statement

This reflects my personal experience in several medical programs. No data are presented and neither individuals nor institutions are identified.

## External Funding

This article has not had any External Funding
