# Supplementary material for: Choosing a medical school: Advice for applicants
Source: MedEdPublish (2016). 2020 Aug 4;9:156. [Version 1] doi: 10.15694/mep.2020.000156.1 (PMC10702655; doi:10.15694/mep.2020.000156.1)
Supplement: Peer review history

# Reviewer report: Reviewer response for version 1

**DOI:** 10.21956/mep.20137.r31092

**Published:** 2020-08-13

**Authors:** Ben Canny

**Affiliations:** 1 Monash University

This review has been migrated. The reviewer awarded 5 stars out of 5 This paper has much practical advice, and, importantly, attempts to dismiss, or at least clarify, a number of "urban" myths. Like others, this should be compulsory reading for those contemplating entering medical school, as well as those responsible for medical school admissions/marketing. Thanks Richard.

Reviewer Expertise:

NA

No decision status is available

# Reviewer report: Reviewer response for version 1

**DOI:** 10.21956/mep.20137.r31091

**Published:** 2020-08-13

**Authors:** Tarun Sen Gupta

**Affiliations:** 1 James Cook University

This review has been migrated. The reviewer awarded 5 stars out of 5 This paper is packed with practical advice that is applicable in many jurisdictions - reflecting Richard Hays' extensive experience as an educator, leader and accreditor for a number of medical schools worldwide. Many of these tips will be familiar to those involved in selection processes - but equally unfamiliar to potential applicants who may not have 'inside knowledge' due to their connections. It therefore serves an important role in making available much of this tacit knowledge, and democratizing access to medical education.There are a number of thoughtful observations in this paper about the type of medical schools we have, or aspire to have, and the sort of applicants we should be selecting. Food for thought for all of us.

Reviewer Expertise:

NA

No decision status is available

# Reviewer report: Reviewer response for version 1

**DOI:** 10.21956/mep.20137.r31093

**Published:** 2020-08-08

**Authors:** Jennifer Cleland

**Affiliations:** 1 University of Aberdeen

This review has been migrated. The reviewer awarded 4 stars out of 5 I really enjoyed this article as it was full of common-sense and very useful to potential applicants. An open access forum is ideal for this type of paper, as it needs to get to the right audience - applicants, teachers and parents. Admissions Deans and medical schools will also find this useful. And salutary - I did like the content about "bland" mission statements and accreditation more or less meaning equality!

Reviewer Expertise:

NA

No decision status is available

# Reviewer report: Reviewer response for version 1

**DOI:** 10.21956/mep.20137.r31095

**Published:** 2020-08-06

**Authors:** P Ravi Shankar

**Affiliations:** 1 American International Medical University

This review has been migrated. The reviewer awarded 5 stars out of 5 I enjoyed reading this article on choosing a medical school. The author’s advise is sound and practical. In South Asia most aspects of medical school are centrally regulated. Most admissions take place through centralized exams using single-best multiple-choice questions. Other aspects like aptitude, social commitment and others are not usually assessed. A few schools have greater freedom in choosing their students. There are differences in facilities and academic standards between schools. Some institutions of excellence conduct their own entrance examinations. A major benefit is that these institutions have plenty of seats reserved for their own candidates for postgraduation. Postgraduation remains a major problem for most undergraduates and the competition is fierce. In the Caribbean medical schools mainly targeted toward external students (offshore medical schools) there is usually no entrance examination though some schools require that candidates pass the Medical college admission test (MCAT). Selection is usually based on a personal essay, volunteering experience, academic performance and a personal interview conducted online. North American students have to pass the United States medical licensing exam (USMLE) to practice in their home country. The first time USMLE step 1 pass rate is often used as one of the accreditation standards. Some of the big schools admit 400 to 500 students in an intake. By the time they complete the basic sciences and are ready to appear in the USMLE the number is whittled down. The schools use various mock exams to ensure that their students are ready for the USMLE and will pass the same at the first attempt. The percentage of originally admitted students who pass all required licensing exams and qualify is not usually advertised and is much lower. This may be a good index for a new student when deciding which school to attend. Accrediting bodies are slowly beginning to also look at the attrition rate. I am happy that this article shows up easily on an online search as it will be a useful guide for students considering a career in medicine.

Reviewer Expertise:

NA

No decision status is available

# Reviewer report: Reviewer response for version 1

**DOI:** 10.21956/mep.20137.r31094

**Published:** 2020-08-05

**Authors:** Ian Wilson

**Affiliations:** 1 International Medical University

This review has been migrated. The reviewer awarded 5 stars out of 5 This paper reflects Prof Hays extensive experience and knowledge. there is nothing in this I disagree with. It should be read by all students applying for medicine, all student advisers and all admissions personnel.A excellent paper.

Reviewer Expertise:

NA

No decision status is available
